# Is treatment in certified cancer centers related to better survival in patients with pancreatic cancer? Evidence from a large German cohort study

**DOI:** 10.1186/s12885-022-09731-w

**Published:** 2022-06-07

**Authors:** Martin Roessler, Jochen Schmitt, Christoph Bobeth, Michael Gerken, Kees Kleihues-van Tol, Christoph Reissfelder, Bettina M. Rau, Marius Distler, Pompiliu Piso, Christian Günster, Monika Klinkhammer-Schalke, Olaf Schoffer, Veronika Bierbaum

**Affiliations:** 1 Center for Evidence-Based Healthcare (ZEGV), Faculty of Medicine, University Hospital Carl Gustav Carus and Carl Gustav Carus, TU Dresden, Fetscherstr. 74, 01307 Dresden, Germany; 2 Tumorzentrum Regensburg - Institut für Qualitätssicherung und Versorgungsforschung, Universität Regensburg, Regensburg, Germany; 3 Association of German Tumor Centers (ADT), Berlin, Germany; 4 Department of Surgery, Universitätsmedizin Mannheim, Medical Faculty Mannheim, Heidelberg University, Mannheim, Germany; 5 Department of General, Visceral and Thoracic Surgery, Hospital of Neumarkt, Neumarkt in der Oberpfalz, Germany; 6 Technische Universität Dresden, Faculty of Medicine and University Hospital Carl Gustav Carus, Department of Visceral-, Thoracic and Vascular Surgery, Dresden, Germany; 7 National Center for Tumor Diseases (NCT/UCC), Dresden, Germany; 8 German Cancer Research Center (DKFZ), Heidelberg, Germany; 9 Faculty of Medicine and University Hospital Carl Gustav Carus, Technische Universität Dresden, Dresden, Germany; 10 Helmholtz-Zentrum Dresden - Rossendorf (HZDR), Dresden, Germany; 11 Department of General and Visceral Surgery, Barmherzige Brueder Hospital Regensburg, Regensburg, Germany; 12 AOK Research Institute (WIdO), Berlin, Germany

**Keywords:** Certified cancer center, Pancreatic cancer, Cohort study, Survival, Cox regression

## Abstract

**Background:**

Treatment of cancer patients in certified cancer centers, that meet specific quality standards in term of structures and procedures of medical care, is a national treatment goal in Germany. However, convincing evidence that treatment in certified cancer centers is associated with better outcomes in patients with pancreatic cancer is still missing.

**Methods:**

We used patient-specific information (demographic characteristics, diagnoses, treatments) from German statutory health insurance data covering the period 2009–2017 and hospital characteristics from the German Standardized Quality Reports. We investigated differences in survival between patients treated in hospitals with and without pancreatic cancer center certification by the German Cancer Society (GCS) using the Kaplan–Meier estimator and Cox regression with shared frailty.

**Results:**

The final sample included 45,318 patients with pancreatic cancer treated in 1,051 hospitals (96 GCS-certified, 955 not GCS-certified). 5,426 (12.0%) of the patients were treated in GCS-certified pancreatic cancer centers. Patients treated in certified and non-certified hospitals had similar distributions of age, sex, and comorbidities. Median survival was 8.0 months in GCS-certified pancreatic cancer centers and 4.4 months in non-certified hospitals. Cox regression adjusting for multiple patient and hospital characteristics yielded a significantly lower hazard of long-term, all-cause mortality in patients treated in GCS-certified pancreatic centers (Hazard ratio = 0.89; 95%-CI = 0.85–0.93). This result remained robust in multiple sensitivity analyses, including stratified estimations for subgroups of patients and hospitals.

**Conclusion:**

This robust observational evidence suggests that patients with pancreatic cancer benefit from treatment in a certified cancer center in terms of survival. Therefore, the certification of hospitals appears to be a powerful strategy to improve patient outcomes in pancreatic cancer care.

**Trial registration:**

ClinicalTrials.gov (NCT04334239).

**Supplementary Information:**

The online version contains supplementary material available at 10.1186/s12885-022-09731-w.

## Background

Pancreatic cancer is among the twelve most common cancer types worldwide [1, 2]. In Germany, standardized yearly incidence rates (per 100,000 persons, ESR) in 2016 were 14.4 in men (rank 6) and 10.9 in women (rank 10) [3]. Pancreatic cancer is associated with poor prognosis as it is usually discovered at high UICC stages (UICC: Union for International Cancer Control) and has often metastasized upon first diagnosis [4]. Surgical resection, which is possible in approximately 20% of patients at the time of diagnosis [5], is the only treatment offering potential cure of pancreatic cancer [6].

In line with international progress in the formation of pancreatic cancer centers [7–9], certification of cancer centers is a national healthcare goal in Germany. This goal was formulated in the “Nationaler Krebsplan” of the German Federal Ministry of Health [10]. It is supposed that high structural and procedural quality standards required for certification of cancer centers ensure patient benefit in terms of effectiveness, safety, and treatment outcome. However, the international literature only offers very preliminary evidence for better patient outcomes in certified cancer centers compared to hospitals without such certification [11–13].

In Germany, organ-specific certification programs are mainly offered by the German Cancer Society (GCS; German: Deutsche Krebsgesellschaft, DKG). GCS certification started in 2003 for breast cancer centers [14]. Currently, there are 1038 GCS-certified centers for different types of cancer, including pancreatic cancer. Previous studies provide some evidence for better patient outcomes in GCS certified cancer centers for colorectal and prostate cancer [12, 15–18], and mixed results for breast cancer [19–21]. This evidence is restricted due to limited regional and time coverage, relatively small sample sizes, and missing relevant covariates at patient and hospital level. Specific evidence for pancreatic cancer is still missing.

We investigated differences in survival between patients treated in GCS-certified pancreatic cancer centers and patients treated in non-certified German hospitals hypothesizing that patients benefit from treatment in a certified center. We used a sample of more than 45,000 individuals with incident pancreatic cancer treated between 2009–2017, allowing for operationalization of relevant patient- and hospital-level confounders.

## Methods

### The WiZen study

WiZen is a cohort study based on German routine health insurance data provided by the AOK Research Institute (Wissenschaftliches Institut der AOK, WIdO) and data provided by the cancer registries Dresden, Erfurt, and Regensburg. The main objective of the study is to compare German certified cancer centers and non-certified hospitals regarding patient survival. The study addresses breast cancer, colorectal cancer, gynecological cancer, head and neck cancer, lung cancer, neurooncological tumors, pancreatic cancer, and prostate cancer. Here, we report results for pancreatic cancer based on health insurance data.

The WiZen study combines medical expertise with profound data analysis targeted at health insurance and cancer registry data. Clinical experts contribute to case and variable definitions, selection and definition of outcomes, relevant risk factors, and treatments, and interpretation and discussion of empirical findings. Methodological expertise and experience in preparation and modeling of health insurance data is provided by the Center for Evidence-Based Healthcare (Zentrum für evidenzbasierte Gesundheitsversorgung, ZEGV).

### Data sources

Patient characteristics were derived from AOK health insurance data, covering the period 2006–2017. The data included oncological and non-oncological inpatient and outpatient diagnoses (codes according to International Statistical Classification of Diseases—German Modification; ICD-10-GM), treatments and medical procedures in terms of OPS codes (German adaption of ICMP) and EBM (Einheitlicher Berwertungsmaßstab, the German outpatient procedure coding system), ATC codes (medical prescriptions), dates of hospital admissions and discharges, demographic characteristics (age, sex), insurance status, and date of death. A patient was considered as incident in the period 2009–2017 only if there was no diagnosis of pancreatic cancer in 2006–2008.

We used data on hospital characteristics from the German Standardized Quality Reports (SQR, German: Standardisierte Qualitätsberichte). Publishing these reports is mandatory for all German hospitals. For each calendar year in the data set, we used most recent information from SQR data (2010, 2012, 2014, or 2016). Finally, the GCS provided information on GCS-certified cancer centers, including the exact date of certification.

### Data protection and ethics

Data on GCS certification, patient and hospital characteristics included in hospital insurance data were pseudonymized at WIdO. Pseudonymized data were analyzed at ZEGV. The WiZen study was approved by the ethics committee of the TU Dresden (approval number: EK95022019). The study was registered at ClinicalTrials.gov (identifier: NCT04334239). Data processing and analyses was conducted in line with the General Data Protection Regulation of the European Union.

### Inclusion and exclusion criteria

From the cohort of patients with the first diagnosis of pancreatic cancer (ICD-10-GM: C25) in 2009–2017 we excluded those who a) were not covered by AOK insurance over the entire observation period, b) had no inpatient primary diagnosis of pancreatic cancer, c) were younger than 18 years of age at date of diagnosis, d) died at date of diagnosis, and if e) there were missing hospital characteristics of the treating hospital. In addition, we excluded patients if they f) were treated in a hospital which became a GCS-certified center within 1 year subsequent to treatment as these hospitals are likely to have already established structures required for certification prior to certificate issue, which would introduce potential misclassification bias. A detailed description of all reasons for exclusion is provided in the Additional file 1.

### Outcome

Primary outcome was all-cause mortality. Follow-up for each included patient started at the date of index treatment and ended at date of death or the end of the observation period (December 31,2017), respectively. Index treatment was defined as the first entity-specific inpatient treatment documented in combination with primary ICD-10-GM diagnosis C25. We considered dates of death until December 31, 2017 and treated all other patients as right-censored at this date using the complete approach [22]. Survival time was expressed in years in all statistical analyses.

### Treatment in certified cancer center

The GCS certifies German cancer centers fulfill pre-specified criteria (e.g. adherence to relevant clinical guidelines, minimum number of treated patients per year) [23]. Currently (August 2021), there are 120 GCS-certified pancreatic cancer centers [24], which belong to the group of visceral oncology centers. Since the (quality of) primary resection has relevant influence on survival prospects, we considered a patient to have received treatment in a GCS-certified cancer center if primary tumor resection, as indicated by entity-specific OPS-codes (5–601, 5–602.y, 5–604) in the presence of primary diagnosis C25, was conducted in a GCS-certified pancreatic cancer center. In case of no documented primary resection, we used the first inpatient treatment with primary diagnosis C25 to determine whether the patient was treated in a GCS-certified pancreatic cancer center.

For hospitals that form an association, no 1:1 merge with data on certification was possible. We considered patients who were treated in a hospital belonging to an association to have received center treatment if at least one of the hospitals belonging to that association was GCS-certified. The rationales for this decision were that 1) there may be spill-over of expertise between certified and non-certified hospitals forming an association and 2) treating non-certified hospitals as certified results in a rather conservative estimation of the certification effect, implying that the true effect may be larger in absolute terms. In addition, we stratified single hospitals and hospitals forming an association for sensitivity analysis.

### Covariates

At the patient level, we adjusted for age at index treatment, sex, the presence of distant metastases (ICD-10-GM: C78-C79) and other oncological diseases prior to/at first diagnosis of pancreatic cancer, and 17 Elixhauser comorbidities [25] selected by clinical experts in the study team (a detailed description of covariates is provided in the Additional file 1, Table S1). Elixhauser comorbidities were included as separate, binary covariates. At the hospital level, we used data on the number of hospitals beds, university hospital status, teaching hospital status, and hospital ownership (public/non-profit/private). In addition, we included dummies for the calendar year of index treatment in all model specifications. These dummies capture potential effects of medical progress and imperfect washout at beginning of the observation period.

### Statistical methods

We described patient and hospital characteristics by median and first/third quartile (Q1/Q3) in case of continuous variables and absolute and relative frequencies in case of categorical variables. Analysis of patient survival is subject to multiple methodological challenges [26, 27]. Accordingly, multiple methodological approaches for survival analysis have been proposed [28]. To estimate differences in unadjusted survival between GCS-certified pancreatic cancer centers and non-certified hospitals in the first five years after index treatment, we applied the Kaplan–Meier estimator as a well-established, non-parametric method [28]. We adjusted differences in total patient survival between GCS-certified pancreatic cancer centers and non-certified hospitals for patient and hospital characteristics using Cox regression with shared frailty [29]. Covariates were included in the Cox models to get as close as possible to the cause-specific survival rate despite the non-randomized study design. Compared with fully parametric survival models, the Cox regression model offers the advantage that the baseline hazard does not have to be specified for consistent estimation of the model coefficients [29]. Our large sample including more than 45,000 patients provided a sound basis for exploiting this statistical property of consistency. By including a random intercept at the hospital level, the Cox model with shared frailty accounted for correlation between outcomes of patients treated in the same hospital [29].

### Sensitivity analyses

For sensitivity analysis, we estimated separate Cox models for specific patient and hospital groups (sex (male/female), other oncological disease (yes/no), single hospital/association, distant metastasis (yes/no), resection (yes/no), number of hospital beds (< 500, >  = 500)). In addition, we explored differences between GCS-certified pancreatic cancer centers according to continuity of certification. To assess the robustness of our results regarding alternative definitions of survival time, we replaced survival since index treatment by survival since first diagnosis for further sensitivity analysis. Furthermore, we censored the survival of all patients one year after index treatment to explore the sensitivity of our results regarding length of follow up. Finally, we excluded patients with incident pancreatic cancer in the most recent data year (2017). The rationale for this analysis was that information on patients’ dates of death included in our data may be less complete in more recent data years due to delays in reporting of deaths to the health insurance. Excluding the most recent data year mitigates the influence of such potentially incomplete information on our findings.

## Results

### Inclusion and exclusion

**Fig. 1:**
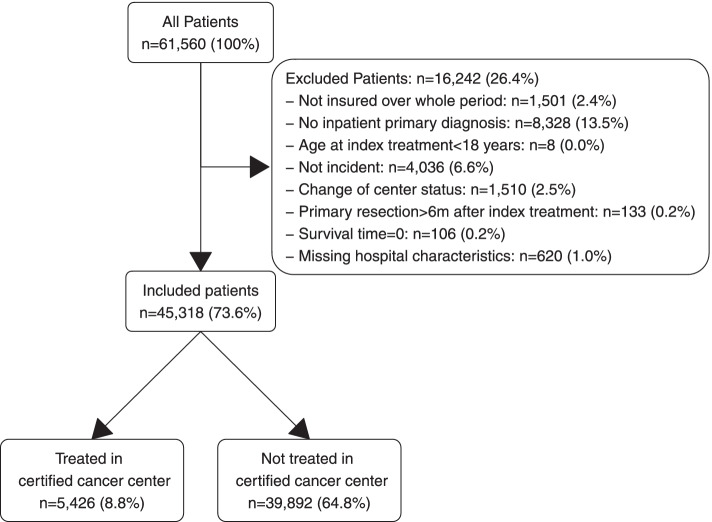
Inclusion and exclusion of patients

From a total of 61,560 patients with pancreatic cancer, 45,318 (73.6%) met our predefined eligibility criteria and were included in the study (Fig. 1). 5,426 (12.0%) of the included patients were treated in GCS-certified pancreatic cancer centers.

### Treatment in GCS-certified pancreatic cancer centers over time

**Fig. 2:**
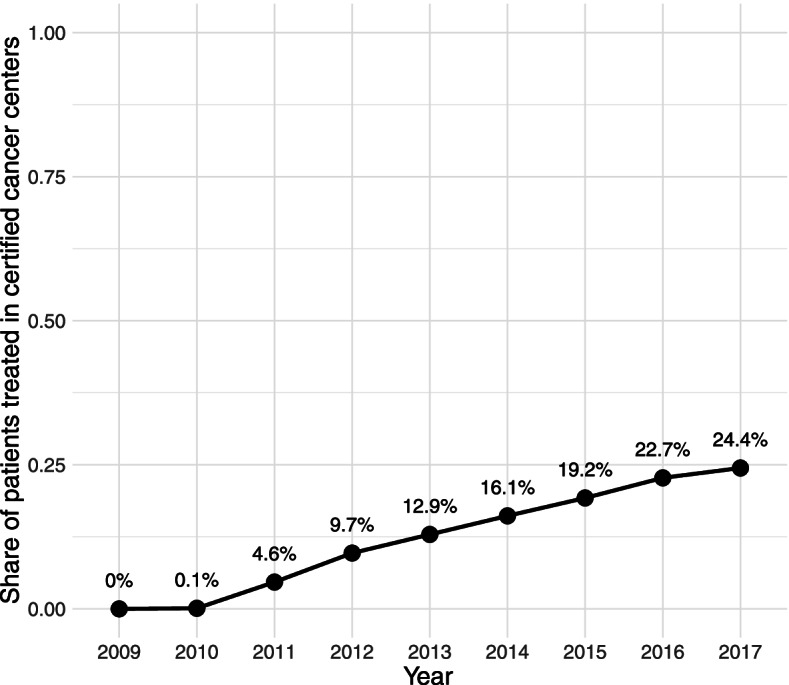
Share of patients treated in GCS-certified pancreatic cancer centers over time

The share of patients treated in GCS-certified pancreatic cancer centers increased steadily during the observation period (Fig. 2). To the extent that medical progress led to better survival of patients with pancreatic cancer in more recent years, the resulting correlation with the share of patients treated in GCS-certified pancreatic cancer centers would induce bias in the estimator of the certification effect. As outlined in the Methods section, all of our models therefore adjust for the year of index treatment to mitigate this potential bias.

### Patient and hospital characteristics

Patients treated in certified centers were characterized by a lower percentage of distant metastases (48.4% vs. 54.3%) and a higher percentage of other oncological diseases (37.3% vs. 35.5%) (Table 1). There were no relevant differences regarding other comorbidities (not shown in the table, the full descriptive statistics are provided in Additional file 1, Table S2) and demographic characteristics. The share of patients with tumor resection was higher in GCS-certified pancreatic cancer centers compared with non-certified hospitals. GCS-certified centers were generally characterized by higher number of beds and higher shares of teaching, university, and public hospitals (Table 2).

**Table 1:** Patient characteristics

Variable	Certified: no	(*n* = 39,892)	Certified: yes	(*n* = 5,426)
Age in years, Median (Q1;Q3)	74	(67;81)	73	(64;79)
Sex, *n* (%)				
female	20,859	(52.3%)	2,754	(50.8%)
male	19,033	(47.7%)	2,672	(49.2%)
Distant metastasis, *n* (%)				
no	18,234	(45.7%)	2,799	(51.6%)
yes	21,658	(54.3%)	2,627	(48.4%)
Resection, *n* (%)				
no	31,715	(79.5%)	3,432	(63.3%)
yes	8,177	(20.5%)	1,994	(36.7%)

Elixhauser comorbidities are not shown in the table. The full results are provided in Additional file 1, Table S2

**Table 2:** Hospital characteristics

Variable	Certified: no	(*n* = 955)	Certified: yes	(*n* = 96)
Hospital beds, *n* (%)				
1–299	573	(60%)	2	(2.1%)
300–499	250	(26.2%)	18	(18.8%)
500–999	112	(11.7%)	46	(47.9%)
1000 +	20	(2.1%)	30	(31.2%)
Teaching hospital, *n* (%)				
no	419	(43.9%)	19	(19.8%)
yes	536	(56.1%)	77	(80.2%)
University hospital, *n* (%)				
no	944	(98.8%)	78	(81.2%)
yes	11	(1.2%)	18	(18.8%)
Hospital ownership, *n* (%)				
public	314	(32.9%)	65	(67.7%)
non-profit	443	(46.4%)	19	(19.8%)
private	198	(20.7%)	12	(12.5%)

### Survival

**Fig. 3:**
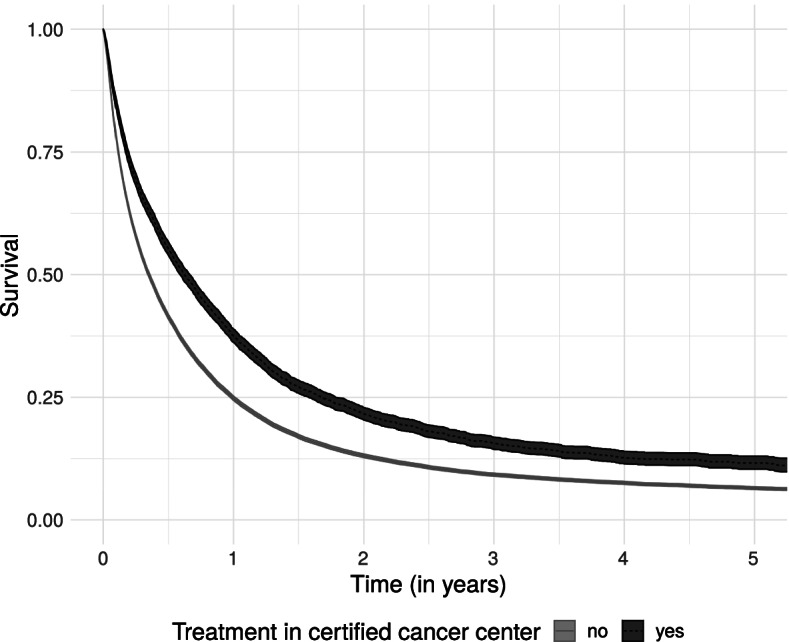
Survival by center status with 95%-confidence intervals

Kaplan–Meier estimates indicated better survival of patients treated in GCS-certified pancreatic cancer centers (Fig. 3). The median survival time was 8.0 months in GCS-certified pancreatic cancer centers and 4.4 months in non-certified hospitals.

The estimated five-year survival proportion was 0.12 (95%-CI = 0.10–0.13) in GCS-certified and 0.06 (95%-CI = 0.06–0.07) in non-certified hospitals.

### Main regression results

When adjusting for the patient’s year of index treatment, the hazard ratio (HR) for GCS-certified pancreatic cancer centers also indicated significantly better survival of patients treated in those centers relative to non-certified hospitals (HR = 0.82; 95%-CI = 0.78–0.86) (Table 3).

**Table 3:** Hazard ratios (HRs) and 95%-confidence intervals (CI) from Cox regression with shared frailty

Variable	HR	CI	HR	CI	HR	CI	HR	CI
Certified center (ref: no)	-	-	-	-	-	-	-	-
yes	0.82	(0.78,0.86)	0.82	(0.78,0.86)	0.84	(0.81,0.88)	0.89	(0.85,0.93)
Age (ref: 18-59)	-	-	-	-	-	-	-	-
60-79			1.43	(1.38,1.48)	1.47	(1.42,1.52)	1.46	(1.41,1.51)
80+			2.22	(2.14,2.31)	2.49	(2.39,2.59)	2.46	(2.37,2.56)
Sex (ref: female)	-	-	-	-	-	-	-	-
male			1.05	(1.03,1.07)	1.04	(1.02,1.06)	1.04	(1.02,1.06)
Other oncological disease (ref: no)	-	-	-	-	-	-	-	-
yes			0.94	(0.92,0.96)	0.85	(0.83,0.87)	0.85	(0.83,0.87)
Distant metastasis (ref: no)	-	-	-	-	-	-	-	-
yes					2.33	(2.28,2.38)	2.31	(2.27,2.36)
Hospital beds (ref: 1-299)	-	-	-	-	-	-	-	-
300-499							0.95	(0.92,0.99)
500-999							0.88	(0.84,0.92)
1000+							0.82	(0.77,0.87)
Teaching hospital (ref: no)	-	-	-	-	-	-	-	-
yes							0.96	(0.93,0.99)
University hospital (ref: no)	-	-	-	-	-	-	-	-
yes							0.81	(0.74,0.88)
Hospital ownership (ref: public)	-	-	-	-	-	-	-	-
non-profit							1.00	(0.96,1.03)
private							1.01	(0.97,1.05)
Calendar year dummies	yes		yes		yes		yes	
Elixhauser comorbidities	no		yes		yes		yes	
Number of patients	45,318		45,318		45,318		45,318	
Number of hospitals	1,051		1,051		1,051		1,051	
SD(RE)	0.26		0.21		0.19		0.16	

*HR* Hazard ratio, *CI* = 95%-confidence interval, *SD *(RE) Standard deviation of the random intercept. Calendar year dummies and Elixhauser comorbidities are included in the regression but not shown in the table. The full regression table is shown in the Additional file 1 (Table S3)

Adjustment for demographic characteristics and comorbidities did not induce changes in the estimated hazard ratio (HR = 0.82; 95%-CI = 0.78–0.86). Survival prospects were worse for older patients and male patients. In addition, there was evidence for better survival of patients with other oncological diseases diagnosed prior to or simultaneously with pancreatic cancer.

Inclusion of distant metastasis as a covariate reduced the estimated center effect (in absolute terms) (HR = 0.84; 95%-CI = 0.81–0.88), which reflects that case mix was more favorable in GCS-certified pancreatic cancer centers.

Adjustment for hospital characteristics further reduced of the estimated center effect (HR = 0.89; 95%-CI = 0.85–0.93). This model specification provided evidence for better survival prospects of patients treated in hospitals with a higher number of beds and university hospitals. In addition, there was modest evidence for better survival of patients treated in teaching hospitals. Differences according to type of hospital ownership (public/non-profit/private) were not observed.

### Results of sensitivity analyses

The estimation results remained robust against stratification by sex (male/female), other oncological disease (yes/no), single hospital/association, distant metastasis (yes/no), tumor resection (yes/no), and number of hospital beds (< 500, >  = 500) (Additional file 1, Table S4). In addition, we found that the certification effect was more pronounced in GCS-certified centers with a longer continuity of certification (Additional file 1, Tables S5 and S6): While the estimated hazard ratio for pancreatic cancer centers certified less than 1 year was 0.91 (95%-CI = 0.85–0.97), the hazard ratio was 0.77 (95%-CI = 0.68–0.87) for centers certified for 5 or more years.

Further sensitivity analyses (Additional file 1, Table S7) indicated that replacing survival time since index treatment by survival time since first diagnosis resulted in a similar estimate of the certification effect (HR = 0.87; 95%-CI = 0.83–0.91). The same was true when censoring follow-up after one year for all patients (HR = 0.88; 95%-CI = 0.84–0.92) and when excluding patients with incident pancreatic cancer in 2017 (HR = 0.89; 95%-CI = 0.85–0.93).

## Discussion

Through use of a broad dataset covering more than 45,000 patients treated in 1,051 hospitals within the period 2009–2017, this study provides new and important evidence that treatment in GCS-certified pancreatic cancer centers is related to better survival in patients with pancreatic cancer. According to our full model, the hazard of death was 11% lower in patients treated in GCS-certified pancreatic cancer centers relative to patients treated in non-certified hospitals, which implies a relevant survival benefit. The results remained robust in several sensitivity analyses. Sensitivity analyses further indicated that the positive effects of certification on patient survival even appear to increase over time. The hazard of death was 23% lower in patients treated in a cancer center certified for 5 or more years compared to patients treated in a non-certified hospital. These results have a high public health impact, as most patients with pancreatic cancer in Germany are still treated in non-certified hospitals. Our findings are also important on a health services research and healthcare management level as they indicate that a complex quality assurance program with a focus on structural and procedural quality such as cancer center certification can have measurable positive effects on patient outcome according to the principles of evidence-based healthcare.

### Strengths and limitations

Our study has several important strengths and extends previous research on cancer center certification. Our broad and unselected sample of patients from all over Germany provides robust and reliable evidence on survival benefits of incident patients with pancreatic cancer treated in certified cancer centers. Given difficulties related to recruitment of study participants from this seriously ill population, the high number of included patients and the long observation period of 11 years are main advantages of our study. The survival of patients in our sample was similar to the survival of patients with pancreatic cancer reported based on epidemiological data from German cancer registries [3]. Our results are also consistent with the (net survival) estimates reported for Germany in the EUROCARE-5 study [30]. These similarities further support the validity and generalizability of our results. Due to limited availability of randomized controlled trials, our comprehensive analysis represents one of the most reliable available sources of real-world evidence on cancer center certification.

We considered all patients who were treated in a hospital belonging to an association to have received center treatment if at least one hospital belonging to that association was GCS-certified. As outlined above, this implies that the true survival differences between patients treated in certified pancreatic cancer centers and non-certified hospitals may be larger than estimated based on our data. The strength and robustness of our findings is further underlined by the results of comprehensive sensitivity analyses. Notably, these analyses indicated a positive relationship between GCS-certified pancreatic cancer center status and survival also in patients with distant metastasis.

The use of observational data generally requires strong assumptions (e.g. complete adjustment for relevant covariates, no reverse causality) for a cause-relationship interpretation of the results. These assumptions are not empirically testable. With this limitation, our study is generally in line with all studies on certification of cancer centers and complex interventions targeted at quality improvement. However, adjustment for many relevant patient and hospital characteristics indicates validity and robustness of our findings.

Another limitation is that our analysis included data from a single German health insurance, which covers approximately 30% of all insured persons in Germany. Hence, inclusion of total patient volume at the hospital level as a covariate was not possible. Since a minimum case volume is part of the requirements for GCS certification, the difference between GCS-certified pancreatic cancer centers and non-certified hospitals may in part be due to differences in case volume [31].

While we adjusted for disease severity using information distant metastases, more detailed information on cancer stage was not available in our data. Potentially unobserved differences between patients treated in certified pancreatic cancer centers and patients treated in non-certified hospitals regarding the distribution of tumor stages therefore could not be included in our regression models. Moreover, the observed differences in case mix between GCS-certified pancreatic cancer centers and non-certified hospitals regarding the share of patients with distant metastasis may indicate selective referral. From a statistical perspective, selective referral would imply endogeneity of treatments in certified cancer centers and induce bias in the estimator of the certification effect.

Another potential source of distortion are incomplete death notifications, which may lead to biased survival estimates. While we cannot exclude such bias, there is some evidence that mortality information in German statutory health insurance data is very valid and complete [32]. Our analysis may also be subject to immortal time bias [33]. However, time from date of diagnosis to index treatment was short and similar for both patients treated in certified pancreatic cancer centers and patients treated in non-certified hospitals (see Additional file 1).

## Conclusions

Our analysis provides robust evidence for better survival prospects of patients with pancreatic cancer treated in GCS-certified pancreatic cancer centers relative to patients treated in non-certified hospitals. In summary, our findings extend and underline previous evidence on effects of treatment in certified cancer centers in general and provide novel evidence for better outcomes in patients with pancreatic cancer. Better survival may result from various aspects such as an adequate and guide-line adherent treatment [5, 6, 34–36]. Further investigation is required to reveal the specific mechanisms behind the relationship between patient outcomes and cancer center status.

## Supplementary Information

**Additional file 1.** 

## Data Availability

The data that support the findings of this study are available from WIdO but restrictions apply to the availability of these data, which were used under license for the current study, and so are not publicly available. Aggregate data are however available from the authors.

## Abbreviations

CI: Confidence interval
GCS: German Cancer Society (Deutsche Krebsgesellschaft, DKG)
HR: Hazard ratio
ICD: International Statistical Classification of Diseases
SQR: German Standardized Quality Reports, Standardisierte Qualitätsberichte
UICC: Union for International Cancer Control
WidO: Wissenschaftliches Institut der AOK, AOK Research Institute
ZEGV: Center for Evidence-based Healthcare
