# Supplementary material for: Is treatment in certified cancer centers related to better survival in patients with pancreatic cancer? Evidence from a large German cohort study
Source: BMC Cancer. 2022 Jun 7;22:621. doi: 10.1186/s12885-022-09731-w (PMC9172168; doi:10.1186/s12885-022-09731-w)
Supplement: Supplementary file 1 — Additional file 1. [file 12885_2022_9731_MOESM1_ESM.pdf]

Additional file 1: Supplementary material

Is treatment in certified cancer centers related to better survival in patients with pancreatic cancer? Evidence from a large German cohort study

Martin Roessler[a], Jochen Schmitt[a], Christoph Bobeth[a], Michael Gerken[b], Kees Kleihues-van Tol[c], Christoph Reissfelder[d], Bettina M. Rau[e], Marius Distler[f,g], Pompiliu Piso[h], Christian Günster[i], Monika Klinkhammer-Schalke[b], Olaf Schoffer[a], Veronika Bierbaum[a]

[a]Center for Evidence-Based Healthcare (ZEGV), University Hospital Carl Gustav Carus and Carl Gustav Carus Faculty of Medicine, TU Dresden, Dresden, Germany
[b]Tumorzentrum Regensburg - Institut für Qualitätssicherung und Versorgungsforschung der Universität Regensburg, Regensburg, Germany
[c]Association of German Tumor Centers (ADT), Berlin, Germany
[d]Department of Surgery, Universitätsmedizin Mannheim, Medical Faculty Mannheim, Heidelberg University, Mannheim, Germany
[e]Department of General, Visceral and Thoracic Surgery, Hospital of Neumarkt, Neumarkt in der Oberpfalz, Germany
[f]Technische Universität Dresden, Faculty of Medicine and University Hospital Carl Gustav Carus, Department of Visceral-, Thoracic and Vascular Surgery,
[g]National Center for Tumor Diseases (NCT/UCC), Dresden, Germany: German Cancer Research Center (DKFZ), Heidelberg, Germany; Faculty of Medicine and University Hospital Carl Gustav Carus, Technische Universität Dresden, Dresden, Germany; Helmholtz-Zentrum Dresden - Rossendorf (HZDR), Dresden, Germany
[h]Department of General and Visceral Surgery, Barmherzige Brueder Hospital Regensburg, Regensburg, Germany
[i]AOK Research Institute (WIdO), Berlin, Germany

Exclusion criteria

In a first step, the following criteria were used by WIdO to select insured persons potentially eligible to be included in the analyses: Ensured with the AOK for at least one day within the period 2006-2017; German postal code; At least one diagnosis of pancreatic cancer (ICD-10-GM: C25) within the period 2006-2017.

In a second step, the following sequential exclusion criteria (with rationales) were applied:

1. Not insured the over whole period (Relevant diagnoses, treatments, and events (incl. death) may not be observed)
2. No primary inpatient diagnosis (The primary inpatient diagnosis indicates entity-specific treatment in a hospital; the focus of the analysis was on survival differences between patients with center and non-center treatments)
3. Age < 18 years (Analysis was restricted to the adult population to exclude rare, special cases of pancreatic cancer)
4. Washout: No inpatient or outpatient diagnosis of pancreatic cancer in the period 2006-2008 (Used for identification of incident cases of pancreatic cancer)
5. Treatment in a hospital which became a certified center within 1 year (Hospitals are likely to have already established structures required for certification before the certificate is issued; patients treated in this 1-year period before certification therefore are likely to have effectively received center treatment)

6. Primary resection more than 6 months after index treatment (Primary tumor resection was used for definition of index treatment only if it occurs within a reasonable time frame after the first relevant inpatient diagnosis of pancreatic cancer; other cases may have low plausibility)
7. Survival time of zero (patients dying at the date of index treatment may not benefit from treatment in certified center)
8. Missing hospital characteristics (The full regression model adjusts for hospital characteristics that represent potential confounders of center treatment. Patients treated in hospitals for which those characteristics were unknown were excluded to ensure comparability of results between different model specifications, i.e. complete case analysis)
9.

Time from first diagnosis to index treatment

Since survival time in our main analysis was defined as time since index treatment, one concern is the presence of immortal time bias. While we cannot exclude such bias, our data shows that the majority of pancreatic cancer patients was treated immediately after diagnosis in both certified pancreatic cancer centers and non-certified hospitals. 90% of all patients received index treatment within 33 days after diagnosis in pancreatic cancer centers and within 41 days in non-certified hospitals.

Supplementary tables

Table S1: Covariates and data sources

Covariate	Description	Data.source
Age	Age at diagnosis (in years)	AOK health insurance data
Sex	Persons' sex as documented in health insurance data (male/female)	AOK health insurance data
Distant metastasis	Primary or secondary inpatient or outpatient diagnosis of distant metastasis (ICD-10-GM: C77-C79) prior to or at index treatment (no/yes)	AOK health insurance data
Other oncological disease	Primary or secondary inpatient or outpatient diagnosis of other oncological disease (ICD-10-GM: C without C25, C77-C79) prior to or at index treatment (no/yes)	AOK health insurance data
Congestive heart failure	Primary or secondary inpatient or outpatient diagnosis of congestive heart failure according to Elixhauser et al. (1998) (yes/no)	AOK health insurance data
Cardiac arrhythmias	Primary or secondary inpatient or outpatient diagnosis of cardiac arrhythmias according to Elixhauser et al. (1998) (yes/no)	AOK health insurance data
Valvular disease	Primary or secondary inpatient or outpatient diagnosis of valvular disease according to Elixhauser et al. (1998) (yes/no)	AOK health insurance data
Pulmonary circulation disorders	Primary or secondary inpatient or outpatient diagnosis of pulmonary circulation disorders according to Elixhauser et al. (1998) (yes/no)	AOK health insurance data
Periph. vascular disorders	Primary or secondary inpatient or outpatient diagnosis of periph. vascular disorders according to Elixhauser et al. (1998) (yes/no)	AOK health insurance data
Hypertension, (uc)	Primary or secondary inpatient or outpatient diagnosis of hypertension, (uncomplicated) according to Elixhauser et al. (1998) (yes/no)	AOK health insurance data
Hypertension, (c)	Primary or secondary inpatient or outpatient diagnosis of hypertension, (complicated) according to Elixhauser et al. (1998) (yes/no)	AOK health insurance data
Other neurological disorders	Primary or secondary inpatient or outpatient diagnosis of other neurological disorders according to Elixhauser et al. (1998) (yes/no)	AOK health insurance data
Chronic pulmonary disease	Primary or secondary inpatient or outpatient diagnosis of chronic pulmonary disease according to Elixhauser et al. (1998) (yes/no)	AOK health insurance data
Diabetes (uc)	Primary or secondary inpatient or outpatient diagnosis of diabetes (uncomplicated) according to Elixhauser et al. (1998) (yes/no)	AOK health insurance data
Diabetes (c)	Primary or secondary inpatient or outpatient diagnosis of diabetes (complicated) according to Elixhauser et al. (1998) (yes/no)	AOK health insurance data

Renal failure	Primary or secondary inpatient or outpatient diagnosis of renal failure according to Elixhauser et al. (1998) (yes/no)	AOK health insurance data
Liver disease	Primary or secondary inpatient or outpatient diagnosis of liver disease according to Elixhauser et al. (1998) (yes/no)	AOK health insurance data
Blood loss anemia	Primary or secondary inpatient or outpatient diagnosis of blood loss anemia according to Elixhauser et al. (1998) (yes/no)	AOK health insurance data
Deficiency anemia	Primary or secondary inpatient or outpatient diagnosis of deficiency anemia according to Elixhauser et al. (1998) (yes/no)	AOK health insurance data
Alcohol abuse	Primary or secondary inpatient or outpatient diagnosis of alcohol abuse according to Elixhauser et al. (1998) (yes/no)	AOK health insurance data
Drug abuse	Primary or secondary inpatient or outpatient diagnosis of drug abuse according to Elixhauser et al. (1998) (yes/no)	AOK health insurance data
Hospital beds	Number of hospital beds (1-299, 300-499, 500-999, 1000+)	German Standardized Quality Reports
Teaching hospital	Hospital has teaching hospital status (yes/no)	German Standardized Quality Reports
University hospital	Hospital has university hospital status (yes/no)	German Standardized Quality Reports
Hospital ownership	Type of hospital ownership (public/non-profit/private) reported	German Standardized Quality Reports

Elixhauser, A., Steiner, C., Harris, D. R., & Coffey, R. M. (1998). Comorbidity measures for use with administrative data. Medical Care, 36(1): 8-27. Note: Outpatient diagnoses were considered only if they were documented in at least two of four successive quarters.

Table S2: Full descriptive statistics

Variable	Certified: no	(n=39,892)	Certified: yes	(n=5,426)
Age in years, Median (Q1;Q3)	74	(67;81)	73	(64;79)
Sex, n (%)				
female	20,859	(52.3%)	2,754	(50.8%)
male	19,033	(47.7%)	2,672	(49.2%)
Congestive heart failure, n(%)				
no	25,763	(64.6%)	3,629	(66.9%)
yes	14,129	(35.4%)	1,797	(33.1%)
Cardiac arrhythmias, n(%)				
no	24,947	(62.5%)	3,239	(59.7%)
yes	14,945	(37.5%)	2,187	(40.3%)
Valvular disease , n(%)				
no	31,809	(79.7%)	4,199	(77.4%)
yes	8,083	(20.3%)	1,227	(22.6%)
Pulmonary circulation disorders, n(%)				
no	36,335	(91.1%)	4,907	(90.4%)
yes	3,557	(8.9%)	519	(9.6%)
Periph. vascular disorders, n(%)				
no	25,889	(64.9%)	3,370	(62.1%)
yes	14,003	(35.1%)	2,056	(37.9%)
Hypertension, (uc), n(%)				
no	6,555	(16.4%)	948	(17.5%)
yes	33,337	(83.6%)	4,478	(82.5%)
Hypertension, (c), n(%)				
no	28,427	(71.3%)	3,826	(70.5%)
yes	11,465	(28.7%)	1,600	(29.5%)
Other neurological disorders, n(%)				
no	35,364	(88.6%)	4,825	(88.9%)
yes	4,528	(11.4%)	601	(11.1%)
Chronic pulmonary disease, n(%)				
no	21,905	(54.9%)	2,632	(48.5%)
yes	17,987	(45.1%)	2,794	(51.5%)
Diabetes (uc) , n(%)				
no	18,836	(47.2%)	2,538	(46.8%)
yes	21,056	(52.8%)	2,888	(53.2%)
Diabetes (c), n(%)				
no	28,737	(72%)	3,835	(70.7%)
yes	11,155	(28%)	1,591	(29.3%)
Renal failure, n(%)				
no	29,909	(75%)	3,965	(73.1%)
yes	9,983	(25%)	1,461	(26.9%)
Liver disease, n(%)				
no	25,592	(64.2%)	3,210	(59.2%)
yes	14,300	(35.8%)	2,216	(40.8%)
Blood loss anemia, n(%)				
no	38,631	(96.8%)	5,240	(96.6%)
yes	1,261	(3.2%)	186	(3.4%)
Deficiency anemia, n(%)				
no	34,636	(86.8%)	4,672	(86.1%)
yes	5,256	(13.2%)	754	(13.9%)
Alcohol abuse, n(%)				
no	36,279	(90.9%)	4,922	(90.7%)

yes	3,613	(9.1%)	504	(9.3%)
Drug abuse, n(%)				
no	38,849	(97.4%)	5,280	(97.3%)
yes	1,043	(2.6%)	146	(2.7%)
Other oncological disease, n (%)				
no	25,743	(64.5%)	3,403	(62.7%)
yes	14,149	(35.5%)	2,023	(37.3%)
Distant metastasis, n (%)				
no	18,234	(45.7%)	2,799	(51.6%)
yes	21,658	(54.3%)	2,627	(48.4%)
Resection, n (%)				
no	31,715	(79.5%)	3,432	(63.3%)
yes	8,177	(20.5%)	1,994	(36.7%)

Table S3: Full regression results (including Year of index treatment and Elixhauser comorbidities): Hazard ratios (HRs) and 95%-confidence intervals (CI) from Cox regression with shared frailty

Variable	HR	CI	HR	CI	HR	CI	HR	CI
Certified center (ref: no)	-	-	-	-	-	-	-	-
yes	0.82	(0.78,0.86)	0.82	(0.78,0.86)	0.84	(0.81,0.88)	0.89	(0.85,0.93)
Year of index treatment (ref: 2009)	-	-	-	-	-	-	-	-
2010	1.03	(0.99,1.07)	1.00	(0.97,1.05)	0.98	(0.94,1.02)	0.98	(0.94,1.02)
2011	1.01	(0.97,1.05)	0.99	(0.95,1.03)	0.96	(0.92,1.00)	0.95	(0.92,0.99)
2012	1.01	(0.97,1.06)	0.98	(0.94,1.02)	0.97	(0.93,1.01)	0.96	(0.92,1.00)
2013	1.04	(1.00,1.09)	1.00	(0.95,1.04)	0.97	(0.93,1.01)	0.96	(0.92,1.00)
2014	1.02	(0.98,1.06)	0.98	(0.94,1.02)	0.95	(0.91,0.99)	0.94	(0.90,0.98)
2015	1.02	(0.97,1.06)	0.96	(0.92,1.00)	0.91	(0.87,0.95)	0.90	(0.86,0.94)
2016	1.02	(0.97,1.06)	0.95	(0.91,1.00)	0.89	(0.85,0.93)	0.88	(0.84,0.92)
2017	1.02	(0.97,1.07)	0.94	(0.89,0.99)	0.88	(0.84,0.93)	0.87	(0.83,0.92)
Age (ref: 18-59)	-	-	-	-	-	-	-	-
60-79			1.43	(1.38,1.48)	1.47	(1.42,1.52)	1.46	(1.41,1.51)
80+			2.22	(2.14,2.31)	2.49	(2.39,2.59)	2.46	(2.37,2.56)
Sex (ref: female)	-	-	-	-	-	-	-	-
male			1.05	(1.03,1.07)	1.04	(1.02,1.06)	1.04	(1.02,1.06)
Congestive heart failure (ref: no)	-	-	-	-	-	-	-	-
yes			1.15	(1.12,1.18)	1.15	(1.13,1.18)	1.15	(1.12,1.18)
Cardiac arrhythmias (ref: no)	-	-	-	-	-	-	-	-
yes			0.97	(0.95,0.99)	0.98	(0.96,1.01)	0.98	(0.96,1.01)
Valvular disease (ref: no)	-	-	-	-	-	-	-	-
yes			0.97	(0.95,1.00)	0.98	(0.96,1.01)	0.99	(0.96,1.01)
Pulmonary circulation disorders (ref: no)	-	-	-	-	-	-	-	-
yes			1.24	(1.20,1.29)	1.18	(1.14,1.22)	1.18	(1.14,1.22)
Periph. vascular disorders (ref: no)	-	-	-	-	-	-	-	-
yes			1.07	(1.04,1.09)	1.06	(1.04,1.08)	1.06	(1.04,1.08)
Hypertension, (uc) (ref: no)	-	-	-	-	-	-	-	-
yes			0.98	(0.95,1.01)	1.01	(0.98,1.04)	1.00	(0.97,1.04)
Hypertension, (c) (ref: no)	-	-	-	-	-	-	-	-
yes			0.96	(0.93,0.98)	0.95	(0.93,0.98)	0.95	(0.93,0.98)

Other neurological disorders (ref: no)	-	-	-	-	-	-	-	-
yes			1.16	(1.13,1.20)	1.18	(1.15,1.22)	1.18	(1.15,1.22)
Chronic pulmonary disease (ref: no)	-	-	-	-	-	-	-	-
yes			0.96	(0.94,0.98)	0.96	(0.94,0.98)	0.96	(0.94,0.98)
Diabetes (uc) (ref: no)	-	-	-	-	-	-	-	-
yes			0.97	(0.95,1.00)	0.99	(0.97,1.02)	0.99	(0.97,1.02)
Diabetes (c) (ref: no)	-	-	-	-	-	-	-	-
yes			1.06	(1.04,1.09)	1.05	(1.02,1.07)	1.05	(1.02,1.07)
Renal failure (ref: no)	-	-	-	-	-	-	-	-
yes			1.12	(1.09,1.15)	1.15	(1.12,1.18)	1.15	(1.12,1.18)
Liver disease (ref: no)	-	-	-	-	-	-	-	-
yes			1.01	(0.98,1.03)	1.00	(0.98,1.03)	1.00	(0.98,1.03)
Blood loss anemia (ref: no)	-	-	-	-	-	-	-	-
yes			1.07	(1.01,1.14)	1.08	(1.02,1.14)	1.08	(1.02,1.14)
Deficiency anemia (ref: no)	-	-	-	-	-	-	-	-
yes			1.04	(1.01,1.08)	1.04	(1.01,1.07)	1.04	(1.01,1.07)
Alcohol abuse (ref: no)	-	-	-	-	-	-	-	-
yes			1.14	(1.10,1.18)	1.16	(1.12,1.21)	1.16	(1.12,1.20)
Drug abuse (ref: no)	-	-	-	-	-	-	-	-
yes			1.00	(0.94,1.06)	1.06	(1.00,1.13)	1.06	(1.00,1.13)
Other oncological disease (ref: no)	-	-	-	-	-	-	-	-
yes			0.94	(0.92,0.96)	0.85	(0.83,0.87)	0.85	(0.83,0.87)
Distant metastasis (ref: no)	-	-	-	-	-	-	-	-
yes					2.33	(2.28,2.38)	2.31	(2.27,2.36)
Hospital beds (ref: 1-299)	-	-	-	-	-	-	-	-
300-499							0.95	(0.92,0.99)
500-999							0.88	(0.84,0.92)
1000+							0.82	(0.77,0.87)
Teaching hospital (ref: no)	-	-	-	-	-	-	-	-
yes							0.96	(0.93,0.99)
University hospital (ref: no)	-	-	-	-	-	-	-	-
yes							0.81	(0.74,0.88)
Hospital ownership (ref: public)	-	-	-	-	-	-	-	-

non-profit							1.00	(0.96,1.03)
private							1.01	(0.97,1.05)
Number of patients	45,318		45,318		45,318		45,318	
Number of hospitals	1,051		1,051		1,051		1,051	
SD(RE)	0.26		0.21		0.19		0.16	

HR=Hazard ratio, CI=95%-confidence interval, SD(RE)=standard deviation of the random intercept.

Table S4: Hazard ratios (HRs) and 95%-confidence intervals (CI) from Cox regression with shared frailty for different strata (Part 1)

Subgroup	Certain insurance status		Sex				Other oncological disease	
	yes		Female		Male		yes	
Variable	HR	CI	HR	CI	HR	CI	HR	CI
Certified center (ref: no)	-	-	-	-	-	-	-	-
yes	0.88	(0.84,0.92)	0.86	(0.80,0.91)	0.90	(0.84,0.95)	0.86	(0.80,0.92)
Year of index treatment (ref: 2009)	-	-	-	-	-	-	-	-
2010	0.98	(0.94,1.02)	0.96	(0.91,1.01)	1.01	(0.95,1.06)	1.01	(0.95,1.09)
2011	0.96	(0.92,1.00)	0.94	(0.88,0.99)	0.97	(0.91,1.03)	0.99	(0.92,1.06)
2012	0.96	(0.92,1.00)	0.95	(0.90,1.01)	0.97	(0.91,1.02)	0.99	(0.92,1.06)
2013	0.96	(0.92,1.00)	0.93	(0.87,0.98)	1.00	(0.94,1.06)	0.96	(0.89,1.03)
2014	0.95	(0.91,0.99)	0.94	(0.89,1.00)	0.94	(0.89,1.00)	0.91	(0.85,0.97)
2015	0.90	(0.87,0.94)	0.91	(0.86,0.96)	0.89	(0.84,0.95)	0.90	(0.84,0.97)
2016	0.88	(0.85,0.93)	0.87	(0.82,0.93)	0.89	(0.84,0.95)	0.87	(0.81,0.94)
2017	0.87	(0.83,0.92)	0.89	(0.83,0.95)	0.86	(0.80,0.92)	0.91	(0.84,0.99)
Age (ref: 18-59)	-	-	-	-	-	-	-	-
60-79	1.44	(1.39,1.49)	1.48	(1.40,1.56)	1.45	(1.39,1.52)	1.41	(1.32,1.50)
80+	2.43	(2.33,2.53)	2.58	(2.43,2.74)	2.34	(2.21,2.47)	2.28	(2.12,2.44)
Sex (ref: female)	-	-	-	-	-	-	-	-
male	1.04	(1.02,1.06)					1.07	(1.03,1.11)
Congestive heart failure (ref: no)	-	-	-	-	-	-	-	-
yes	1.15	(1.12,1.18)	1.16	(1.12,1.20)	1.13	(1.09,1.17)	1.15	(1.10,1.19)
Cardiac arrhythmias (ref: no)	-	-	-	-	-	-	-	-
yes	0.98	(0.96,1.01)	0.98	(0.95,1.01)	1.00	(0.96,1.03)	0.98	(0.95,1.02)
Valvular disease (ref: no)	-	-	-	-	-	-	-	-
yes	0.98	(0.96,1.01)	0.96	(0.92,0.99)	1.02	(0.98,1.07)	1.01	(0.97,1.06)
Pulmonary circulation disorders (ref: no)	-	-	-	-	-	-	-	-
yes	1.17	(1.13,1.22)	1.17	(1.12,1.23)	1.19	(1.13,1.25)	1.13	(1.07,1.20)
Periph. vascular disorders (ref: no)	-	-	-	-	-	-	-	-
yes	1.06	(1.03,1.08)	1.04	(1.01,1.07)	1.08	(1.04,1.11)	1.06	(1.03,1.10)
Hypertension, (uc) (ref: no)	-	-	-	-	-	-	-	-

yes	1.00	(0.97,1.03)	1.00	(0.96,1.05)	1.00	(0.96,1.05)	1.06	(1.00,1.11)
Hypertension, (c) (ref: no)	-	-	-	-	-	-	-	-
yes	0.96	(0.93,0.98)	0.96	(0.93,1.00)	0.95	(0.91,0.99)	0.96	(0.92,1.00)
Other neurological disorders (ref: no)	-	-	-	-	-	-	-	-
yes	1.19	(1.15,1.22)	1.15	(1.10,1.20)	1.22	(1.17,1.28)	1.20	(1.14,1.26)
Chronic pulmonary disease (ref: no)	-	-	-	-	-	-	-	-
yes	0.97	(0.95,0.99)	0.93	(0.91,0.96)	1.00	(0.97,1.03)	0.99	(0.96,1.03)
Diabetes (uc) (ref: no)	-	-	-	-	-	-	-	-
yes	0.99	(0.97,1.02)	1.02	(0.99,1.06)	0.97	(0.93,1.00)	1.01	(0.97,1.05)
Diabetes (c) (ref: no)	-	-	-	-	-	-	-	-
yes	1.04	(1.02,1.07)	1.03	(0.99,1.07)	1.06	(1.02,1.10)	1.03	(0.99,1.08)
Renal failure (ref: no)	-	-	-	-	-	-	-	-
yes	1.15	(1.12,1.18)	1.16	(1.12,1.20)	1.13	(1.09,1.18)	1.14	(1.09,1.19)
Liver disease (ref: no)	-	-	-	-	-	-	-	-
yes	1.01	(0.98,1.03)	0.99	(0.96,1.02)	1.02	(0.99,1.05)	1.00	(0.97,1.04)
Blood loss anemia (ref: no)	-	-	-	-	-	-	-	-
yes	1.08	(1.02,1.14)	1.10	(1.02,1.19)	1.04	(0.95,1.14)	1.01	(0.93,1.11)
Deficiency anemia (ref: no)	-	-	-	-	-	-	-	-
yes	1.04	(1.01,1.08)	1.04	(1.00,1.09)	1.03	(0.99,1.08)	1.03	(0.98,1.08)
Alcohol abuse (ref: no)	-	-	-	-	-	-	-	-
yes	1.16	(1.12,1.21)	1.21	(1.13,1.31)	1.14	(1.09,1.19)	1.19	(1.12,1.27)
Drug abuse (ref: no)	-	-	-	-	-	-	-	-
yes	1.06	(1.00,1.13)	1.10	(1.01,1.19)	1.04	(0.94,1.14)	1.12	(1.01,1.24)
Other oncological disease (ref: no)	-	-	-	-	-	-	-	-
yes	0.85	(0.83,0.87)	0.83	(0.81,0.86)	0.87	(0.85,0.90)		
Distant metastasis (ref: no)	-	-	-	-	-	-	-	-
yes	2.29	(2.24,2.34)	2.30	(2.23,2.37)	2.36	(2.29,2.44)	2.12	(2.05,2.20)
Hospital beds (ref: 1-299)	-	-	-	-	-	-	-	-
300-499	0.95	(0.92,0.99)	0.96	(0.91,1.00)	0.96	(0.91,1.01)	0.98	(0.92,1.03)
500-999	0.88	(0.84,0.92)	0.87	(0.83,0.92)	0.89	(0.84,0.94)	0.87	(0.81,0.93)
1000+	0.82	(0.77,0.88)	0.80	(0.74,0.87)	0.83	(0.77,0.89)	0.77	(0.70,0.84)
Teaching hospital (ref: no)	-	-	-	-	-	-	-	-

yes	0.95	(0.92,0.98)	0.95	(0.91,1.00)	0.95	(0.91,1.00)	0.94	(0.90,0.99)
University hospital (ref: no)	-	-	-	-	-	-	-	-
yes	0.80	(0.73,0.87)	0.82	(0.74,0.91)	0.80	(0.73,0.88)	0.81	(0.72,0.91)
Hospital ownership (ref: public)	-	-	-	-	-	-	-	-
non-profit	0.99	(0.96,1.03)	0.98	(0.94,1.03)	1.01	(0.96,1.05)	0.96	(0.91,1.01)
private	1.00	(0.96,1.05)	1.01	(0.96,1.07)	1.00	(0.94,1.05)	0.97	(0.91,1.04)
Number of patients	43,586		23,613		21,705		16,172	
Number of hospitals	1,050		1,019		1,014		1,005	
SD(RE)	0.16		0.16		0.14		0.19	

Table S4: Hazard ratios (HRs) and 95%-confidence intervals (CI) from Cox regression with shared frailty for different strata (Part 2)

Subgroup			Distance metastasis				Resection			
	no		yes		no		yes		no	
Variable	HR	CI	HR	CI	HR	CI	HR	CI	HR	CI
Certified center (ref: no)	-	-	-	-	-	-	-	-	-	-
yes	0.90	(0.85,0.95)	0.89	(0.84,0.94)	0.86	(0.81,0.92)	0.92	(0.86,0.99)	0.93	(0.88,0.97)
Year of index treatment (ref: 2009)	-	-	-	-	-	-	-	-	-	-
2010	0.96	(0.91,1.01)	0.95	(0.90,1.00)	1.01	(0.95,1.07)	1.00	(0.91,1.10)	1.00	(0.95,1.04)
2011	0.93	(0.88,0.98)	0.94	(0.89,0.99)	0.98	(0.92,1.04)	0.95	(0.87,1.04)	1.00	(0.96,1.04)
2012	0.94	(0.89,0.99)	0.96	(0.91,1.02)	0.96	(0.90,1.01)	0.96	(0.88,1.06)	0.99	(0.94,1.03)
2013	0.96	(0.91,1.01)	0.97	(0.91,1.02)	0.95	(0.89,1.01)	0.95	(0.86,1.05)	1.00	(0.96,1.05)
2014	0.96	(0.91,1.01)	0.95	(0.90,1.01)	0.91	(0.86,0.97)	0.96	(0.87,1.06)	0.97	(0.92,1.01)
2015	0.89	(0.85,0.94)	0.90	(0.85,0.96)	0.89	(0.83,0.95)	0.85	(0.77,0.94)	0.95	(0.91,1.00)
2016	0.88	(0.83,0.93)	0.85	(0.80,0.90)	0.93	(0.87,1.00)	0.91	(0.82,1.02)	0.91	(0.87,0.96)
2017	0.84	(0.79,0.90)	0.88	(0.83,0.94)	0.85	(0.79,0.93)	0.85	(0.74,0.99)	0.92	(0.87,0.97)
Age (ref: 18-59)	-	-	-	-	-	-	-	-	-	-
60-79	1.48	(1.42,1.55)	1.36	(1.31,1.42)	1.63	(1.54,1.72)	1.42	(1.32,1.52)	1.44	(1.38,1.50)
80+	2.59	(2.47,2.72)	2.18	(2.07,2.29)	2.97	(2.79,3.17)	1.99	(1.81,2.19)	2.22	(2.12,2.32)
Sex (ref: female)	-	-	-	-	-	-	-	-	-	-
male	1.02	(0.99,1.05)	1.04	(1.01,1.07)	1.03	(1.00,1.07)	1.09	(1.04,1.15)	1.03	(1.00,1.05)
Congestive heart failure (ref: no)	-	-	-	-	-	-	-	-	-	-
yes	1.16	(1.12,1.19)	1.12	(1.08,1.15)	1.20	(1.15,1.24)	1.11	(1.04,1.17)	1.14	(1.11,1.17)
Cardiac arrhythmias (ref: no)	-	-	-	-	-	-	-	-	-	-
yes	0.98	(0.96,1.01)	0.99	(0.96,1.02)	0.99	(0.95,1.02)	0.99	(0.94,1.05)	0.99	(0.97,1.02)
Valvular disease (ref: no)	-	-	-	-	-	-	-	-	-	-
yes	0.96	(0.93,1.00)	0.99	(0.96,1.03)	0.97	(0.93,1.01)	1.00	(0.93,1.06)	0.99	(0.96,1.02)
Pulmonary circulation disorders (ref: no)	-	-	-	-	-	-	-	-	-	-
yes	1.21	(1.16,1.27)	1.20	(1.15,1.26)	1.16	(1.09,1.23)	1.09	(0.98,1.20)	1.18	(1.13,1.23)

Periph. vascular disorders (ref: no)	-	-	-	-	-	-	-	-	-	-
yes	1.06	(1.03,1.09)	1.05	(1.02,1.08)	1.07	(1.03,1.11)	1.08	(1.02,1.14)	1.05	(1.02,1.07)
Hypertension, (uc) (ref: no)	-	-	-	-	-	-	-	-	-	-
yes	0.98	(0.94,1.01)	1.04	(1.00,1.08)	0.96	(0.91,1.00)	1.00	(0.94,1.07)	0.99	(0.96,1.03)
Hypertension, (c) (ref: no)	-	-	-	-	-	-	-	-	-	-
yes	0.95	(0.92,0.98)	0.96	(0.93,0.99)	0.95	(0.92,0.99)	1.07	(1.00,1.13)	0.93	(0.91,0.96)
Other neurological disorders (ref: no)	-	-	-	-	-	-	-	-	-	-
yes	1.18	(1.13,1.22)	1.17	(1.12,1.22)	1.21	(1.16,1.27)	1.12	(1.02,1.22)	1.16	(1.12,1.20)
Chronic pulmonary disease (ref: no)	-	-	-	-	-	-	-	-	-	-
yes	0.95	(0.92,0.97)	0.97	(0.94,0.99)	0.96	(0.93,0.99)	0.99	(0.95,1.04)	0.96	(0.93,0.98)
Diabetes (uc) (ref: no)	-	-	-	-	-	-	-	-	-	-
yes	0.99	(0.96,1.02)	0.97	(0.94,1.01)	1.02	(0.98,1.06)	1.11	(1.05,1.17)	0.99	(0.97,1.02)
Diabetes (c) (ref: no)	-	-	-	-	-	-	-	-	-	-
yes	1.06	(1.02,1.09)	1.03	(0.99,1.06)	1.08	(1.03,1.12)	0.97	(0.92,1.04)	1.04	(1.01,1.07)
Renal failure (ref: no)	-	-	-	-	-	-	-	-	-	-
yes	1.15	(1.11,1.19)	1.15	(1.11,1.18)	1.16	(1.12,1.20)	1.14	(1.07,1.21)	1.15	(1.12,1.19)
Liver disease (ref: no)	-	-	-	-	-	-	-	-	-	-
yes	1.01	(0.98,1.03)	1.01	(0.98,1.04)	1.00	(0.97,1.03)	1.08	(1.03,1.14)	0.99	(0.96,1.01)
Blood loss anemia (ref: no)	-	-	-	-	-	-	-	-	-	-
yes	1.13	(1.05,1.22)	1.05	(0.97,1.14)	1.11	(1.02,1.21)	1.19	(1.03,1.37)	1.05	(0.98,1.12)
Deficiency anemia (ref: no)	-	-	-	-	-	-	-	-	-	-
yes	1.05	(1.01,1.09)	1.04	(0.99,1.08)	1.05	(1.00,1.10)	0.99	(0.91,1.07)	1.05	(1.01,1.08)
Alcohol abuse (ref: no)	-	-	-	-	-	-	-	-	-	-
yes	1.15	(1.10,1.20)	1.19	(1.13,1.24)	1.14	(1.07,1.21)	1.17	(1.07,1.27)	1.14	(1.10,1.19)
Drug abuse (ref: no)	-	-	-	-	-	-	-	-	-	-
yes	1.02	(0.95,1.11)	1.09	(1.00,1.19)	1.03	(0.94,1.13)	1.04	(0.91,1.19)	1.10	(1.02,1.18)
Other oncological disease (ref: no)	-	-	-	-	-	-	-	-	-	-
yes			0.82	(0.80,0.84)	0.91	(0.88,0.94)	0.88	(0.84,0.93)	0.87	(0.85,0.89)
Distant metastasis (ref: no)	-	-	-	-	-	-	-	-	-	-

yes	2.45	(2.39,2.52)					1.45	(1.36,1.55)	1.92	(1.87,1.97)
Hospital beds (ref: 1-299)	-	-	-	-	-	-	-	-	-	-
300-499	0.94	(0.90,0.98)	0.96	(0.91,1.00)	0.96	(0.91,1.01)	0.95	(0.87,1.04)	1.01	(0.97,1.04)
500-999	0.89	(0.84,0.93)	0.87	(0.83,0.92)	0.90	(0.84,0.96)	0.90	(0.82,0.99)	0.95	(0.91,1.00)
1000+	0.85	(0.78,0.92)	0.81	(0.75,0.87)	0.83	(0.76,0.91)	0.87	(0.79,0.97)	0.92	(0.87,0.98)
Teaching hospital (ref: no)	-	-	-	-	-	-	-	-	-	-
yes	0.96	(0.92,1.00)	0.96	(0.92,1.00)	0.95	(0.90,1.00)	0.97	(0.90,1.04)	1.00	(0.96,1.03)
University hospital (ref: no)	-	-	-	-	-	-	-	-	-	-
yes	0.81	(0.73,0.89)	0.78	(0.70,0.86)	0.85	(0.77,0.95)	0.95	(0.87,1.04)	0.82	(0.76,0.89)
Hospital ownership (ref: public)	-	-	-	-	-	-	-	-	-	-
non-profit	1.02	(0.98,1.06)	0.99	(0.95,1.03)	1.01	(0.97,1.06)	1.05	(0.99,1.12)	0.99	(0.96,1.03)
private	1.03	(0.98,1.08)	1.03	(0.98,1.08)	0.98	(0.92,1.03)	1.10	(1.02,1.19)	0.98	(0.95,1.03)
Number of patients	29,146		24,285		21,033		10,171		35,147	
Number of hospitals	1,027		1,027		1,002		597		1,049	
SD(RE)	0.18		0.15		0.17		0.07		0.13	

Table S4: Hazard ratios (HRs) and 95%-confidence intervals (CI) from Cox regression with shared frailty for different strata (Part 3)

Subgroup	Single location				Hospital beds			
	yes		no		Less than 500		500 or more	
Variable	HR	CI	HR	CI	HR	CI	HR	CI
Certified center (ref: no)	-	-	-	-	-	-	-	-
yes	0.90	(0.85,0.95)	0.80	(0.72,0.88)	0.93	(0.84,1.03)	0.90	(0.85,0.95)
Year of index treatment (ref: 2009)	-	-	-	-	-	-	-	-
2010	0.98	(0.94,1.02)	0.96	(0.86,1.08)	1.00	(0.95,1.06)	0.95	(0.89,1.01)
2011	0.95	(0.91,0.99)	0.99	(0.89,1.10)	0.95	(0.91,1.01)	0.95	(0.89,1.01)
2012	0.95	(0.91,0.99)	1.00	(0.90,1.11)	0.98	(0.93,1.04)	0.93	(0.87,0.99)
2013	0.97	(0.92,1.01)	0.93	(0.84,1.03)	0.99	(0.94,1.04)	0.92	(0.86,0.98)
2014	0.94	(0.89,0.98)	0.94	(0.85,1.04)	0.98	(0.92,1.03)	0.89	(0.84,0.95)
2015	0.90	(0.86,0.94)	0.89	(0.80,0.99)	0.93	(0.88,0.99)	0.86	(0.80,0.92)
2016	0.88	(0.84,0.93)	0.87	(0.78,0.96)	0.91	(0.86,0.97)	0.84	(0.79,0.90)
2017	0.87	(0.82,0.92)	0.87	(0.78,0.98)	0.91	(0.86,0.97)	0.82	(0.76,0.89)
Age (ref: 18-59)	-	-	-	-	-	-	-	-

60-79	1.45	(1.40,1.51)	1.51	(1.40,1.63)	1.49	(1.42,1.57)	1.43	(1.36,1.50)
80+	2.43	(2.32,2.54)	2.61	(2.39,2.85)	2.44	(2.31,2.57)	2.53	(2.38,2.68)
Sex (ref: female)	-	-	-	-	-	-	-	-
male	1.03	(1.01,1.06)	1.06	(1.01,1.11)	1.02	(1.00,1.05)	1.06	(1.03,1.09)
Congestive heart failure (ref: no)	-	-	-	-	-	-	-	-
yes	1.14	(1.11,1.17)	1.18	(1.12,1.25)	1.15	(1.11,1.19)	1.15	(1.11,1.20)
Cardiac arrhythmias (ref: no)	-	-	-	-	-	-	-	-
yes	0.99	(0.96,1.01)	0.98	(0.93,1.02)	0.99	(0.96,1.01)	0.98	(0.95,1.02)
Valvular disease (ref: no)	-	-	-	-	-	-	-	-
yes	0.98	(0.95,1.01)	0.99	(0.94,1.05)	1.00	(0.97,1.04)	0.96	(0.92,1.00)
Pulmonary circulation disorders (ref: no)	-	-	-	-	-	-	-	-
yes	1.18	(1.13,1.23)	1.16	(1.08,1.25)	1.15	(1.10,1.21)	1.22	(1.15,1.29)
Periph. vascular disorders (ref: no)	-	-	-	-	-	-	-	-
yes	1.07	(1.04,1.09)	1.03	(0.99,1.09)	1.04	(1.01,1.07)	1.09	(1.05,1.13)
Hypertension, (uc) (ref: no)	-	-	-	-	-	-	-	-
yes	1.01	(0.98,1.05)	0.98	(0.91,1.04)	0.99	(0.95,1.03)	1.02	(0.97,1.06)
Hypertension, (c) (ref: no)	-	-	-	-	-	-	-	-
yes	0.96	(0.93,0.99)	0.93	(0.88,0.98)	0.95	(0.92,0.99)	0.96	(0.92,0.99)
Other neurological disorders (ref: no)	-	-	-	-	-	-	-	-
yes	1.17	(1.13,1.21)	1.23	(1.15,1.31)	1.22	(1.17,1.27)	1.13	(1.07,1.18)
Chronic pulmonary disease (ref: no)	-	-	-	-	-	-	-	-
yes	0.97	(0.94,0.99)	0.96	(0.91,1.00)	0.96	(0.94,0.99)	0.96	(0.93,0.99)
Diabetes (uc) (ref: no)	-	-	-	-	-	-	-	-
yes	1.00	(0.98,1.03)	0.95	(0.90,1.01)	0.99	(0.96,1.03)	0.99	(0.96,1.03)
Diabetes (c) (ref: no)	-	-	-	-	-	-	-	-
yes	1.03	(1.00,1.07)	1.09	(1.03,1.16)	1.05	(1.01,1.09)	1.04	(1.00,1.09)
Renal failure (ref: no)	-	-	-	-	-	-	-	-
yes	1.15	(1.12,1.19)	1.14	(1.08,1.20)	1.13	(1.10,1.17)	1.17	(1.12,1.21)
Liver disease (ref: no)	-	-	-	-	-	-	-	-
yes	1.02	(0.99,1.04)	0.95	(0.91,1.00)	0.98	(0.95,1.01)	1.04	(1.00,1.07)
Blood loss anemia (ref: no)	-	-	-	-	-	-	-	-
yes	1.07	(1.00,1.14)	1.12	(0.99,1.27)	1.11	(1.03,1.20)	1.04	(0.95,1.14)
Deficiency anemia (ref: no)	-	-	-	-	-	-	-	-

yes	1.04	(1.01,1.08)	1.02	(0.95,1.09)	1.03	(0.99,1.08)	1.04	(0.99,1.09)
Alcohol abuse (ref: no)	-	-	-	-	-	-	-	-
yes	1.17	(1.12,1.22)	1.12	(1.04,1.22)	1.14	(1.08,1.20)	1.19	(1.13,1.26)
Drug abuse (ref: no)	-	-	-	-	-	-	-	-
yes	1.07	(1.00,1.15)	1.03	(0.91,1.18)	1.02	(0.94,1.11)	1.13	(1.03,1.24)
Other oncological disease (ref: no)	-	-	-	-	-	-	-	-
yes	0.86	(0.84,0.88)	0.83	(0.79,0.87)	0.87	(0.85,0.90)	0.82	(0.80,0.85)
Distant metastasis (ref: no)	-	-	-	-	-	-	-	-
yes	2.31	(2.25,2.36)	2.35	(2.24,2.46)	2.25	(2.19,2.32)	2.39	(2.31,2.47)
Hospital beds (ref: 1-299)	-	-	-	-	-	-	-	-
300-499	0.94	(0.90,0.98)	1.00	(0.92,1.08)				
500-999	0.87	(0.83,0.91)	0.90	(0.82,0.99)				
1000+	0.80	(0.74,0.86)	0.91	(0.78,1.05)				
Teaching hospital (ref: no)	-	-	-	-	-	-	-	-
yes	0.96	(0.92,0.99)	0.96	(0.88,1.04)	0.92	(0.89,0.95)	1.04	(0.96,1.12)
University hospital (ref: no)	-	-	-	-	-	-	-	-
yes	0.82	(0.74,0.89)	1.03	(0.80,1.31)	0.80	(0.62,1.03)	0.80	(0.73,0.88)
Hospital ownership (ref: public)	-	-	-	-	-	-	-	-
non-profit	1.00	(0.97,1.04)	0.97	(0.91,1.04)	0.99	(0.95,1.02)	1.05	(0.98,1.12)
private	1.02	(0.97,1.07)	0.94	(0.85,1.04)	1.02	(0.97,1.07)	1.02	(0.94,1.10)
Number of patients	35,547		9,771		25,333		19,985	
Number of hospitals	983		223		860		235	
SD(RE)	0.16		0.13		0.14		0.18	

HR=Hazard ratio, CI=95%-confidence interval, SD(RE)=standard deviation of the random intercept.

Table S5: Number and share of patients treated in GCS-certified cancer centers by continuity of certification

Variable	Certified: yes	(n=5,426)
Continuity of certification, n (%)		
<1 year	1,335	(24.6%)
1-<2 years	1,146	(21.1%)
2-<5 years	2,270	(41.8%)
5 or more years	675	(12.4%)

Table S6: Hazard ratios (HRs) and 95%-confidence intervals (CI) from Cox regression with shared frailty for continuity of GCS-certification

Variable	HR	CI
Continuity of certification (ref: not certified)	-	-
<1 year	0.91	(0.85,0.97)
1-<2 years	0.87	(0.81,0.94)
2-<5 years	0.89	(0.84,0.95)
5 or more years	0.77	(0.68,0.87)
Year of index treatment (ref: 2009)	-	-
2010	0.98	(0.94,1.02)
2011	0.95	(0.91,0.99)
2012	0.96	(0.92,1.00)
2013	0.96	(0.92,1.00)
2014	0.94	(0.90,0.98)
2015	0.90	(0.86,0.94)
2016	0.88	(0.84,0.92)
2017	0.88	(0.84,0.92)
Age (ref: 18-59)	-	-
60-79	1.46	(1.41,1.51)
80+	2.46	(2.37,2.56)
Sex (ref: female)	-	-
male	1.04	(1.02,1.06)
Congestive heart failure (ref: no)	-	-
yes	1.15	(1.12,1.18)
Cardiac arrhythmias (ref: no)	-	-
yes	0.98	(0.96,1.01)
Valvular disease (ref: no)	-	-
yes	0.99	(0.96,1.01)
Pulmonary circulation disorders (ref: no)	-	-
yes	1.18	(1.14,1.22)
Periph. vascular disorders (ref: no)	-	-
yes	1.06	(1.04,1.08)
Hypertension, (uc) (ref: no)	-	-
yes	1.00	(0.97,1.04)
Hypertension, (c) (ref: no)	-	-
yes	0.95	(0.93,0.98)
Other neurological disorders (ref: no)	-	-
yes	1.18	(1.15,1.22)
Chronic pulmonary disease (ref: no)	-	-
yes	0.96	(0.94,0.98)
Diabetes (uc) (ref: no)	-	-
yes	0.99	(0.97,1.02)
Diabetes (c) (ref: no)	-	-
yes	1.05	(1.02,1.07)
Renal failure (ref: no)	-	-
yes	1.15	(1.12,1.18)
Liver disease (ref: no)	-	-
yes	1.00	(0.98,1.03)
Blood loss anemia (ref: no)	-	-
yes	1.08	(1.02,1.14)
Deficiency anemia (ref: no)	-	-
yes	1.04	(1.01,1.07)
Alcohol abuse (ref: no)	-	-

yes	1.16	(1.12,1.20)
Drug abuse (ref: no)	-	-
yes	1.06	(1.00,1.13)
Other oncological disease (ref: no)	-	-
yes	0.85	(0.83,0.87)
Distant metastasis (ref: no)	-	-
yes	2.32	(2.27,2.37)
Hospital beds (ref: 1-299)	-	-
300-499	0.95	(0.92,0.99)
500-999	0.88	(0.84,0.92)
1000+	0.82	(0.77,0.87)
Teaching hospital (ref: no)	-	-
yes	0.96	(0.93,0.99)
University hospital (ref: no)	-	-
yes	0.81	(0.74,0.88)
Hospital ownership (ref: public)	-	-
non-profit	1.00	(0.96,1.03)
private	1.01	(0.97,1.05)
Number of patients	45,318	
Number of hospitals	1,051	
SD(RE)	0.16	

HR=Hazard ratio, CI=95%-confidence interval, SD(RE)=standard deviation of the random intercept.

Table S7: Hazard ratios (HRs) and 95%-confidence intervals (CI) from Cox regression with shared frailty for additional sensitivity analyses

Variable Analysis	HR First diagnosis	CI	HR Censored 1yr	CI	HR Excl. 2017	CI
Certified center (ref: no)	-	-	-	-	-	-
yes	0.87	(0.83,0.91)	0.88	(0.84,0.92)	0.89	(0.85,0.93)
Year of index treatment (ref: 2009)	-	-	-	-	-	-
2010	0.99	(0.95,1.03)	0.97	(0.93,1.02)	0.98	(0.94,1.02)
2011	0.96	(0.92,1.00)	0.95	(0.91,1.00)	0.95	(0.91,0.99)
2012	0.97	(0.93,1.01)	0.96	(0.92,1.00)	0.96	(0.92,1.00)
2013	0.97	(0.93,1.01)	0.94	(0.90,0.99)	0.96	(0.92,1.00)
2014	0.95	(0.91,0.99)	0.92	(0.88,0.97)	0.94	(0.90,0.98)
2015	0.90	(0.86,0.94)	0.89	(0.85,0.93)	0.90	(0.86,0.94)
2016	0.89	(0.85,0.93)	0.87	(0.83,0.91)	0.88	(0.84,0.92)
2017	0.86	(0.82,0.90)	0.86	(0.81,0.90)	NANA	(NA, NA)
Age (ref: 18-59)	-	-	-	-	-	-
60-79	1.47	(1.42,1.52)	1.52	(1.46,1.58)	1.45	(1.40,1.50)
80+	2.43	(2.34,2.53)	2.63	(2.51,2.75)	2.44	(2.34,2.54)
Sex (ref: female)	-	-	-	-	-	-
male	1.04	(1.02,1.06)	1.03	(1.01,1.05)	1.04	(1.02,1.06)
Congestive heart failure (ref: no)	-	-	-	-	-	-
yes	1.14	(1.11,1.17)	1.18	(1.15,1.22)	1.14	(1.11,1.17)
Cardiac arrhythmias (ref: no)	-	-	-	-	-	-
yes	0.98	(0.96,1.00)	0.99	(0.97,1.02)	0.98	(0.96,1.01)
Valvular disease (ref: no)	-	-	-	-	-	-
yes	0.98	(0.95,1.01)	0.98	(0.95,1.01)	0.99	(0.96,1.02)
Pulmonary circulation disorders (ref: no)	-	-	-	-	-	-
yes	1.17	(1.13,1.21)	1.20	(1.16,1.25)	1.18	(1.14,1.23)
Periph. vascular disorders (ref: no)	-	-	-	-	-	-
yes	1.03	(1.01,1.06)	1.06	(1.03,1.08)	1.06	(1.03,1.08)
Hypertension, (uc) (ref: no)	-	-	-	-	-	-
yes	1.00	(0.97,1.03)	1.00	(0.97,1.04)	1.00	(0.97,1.03)
Hypertension, (c) (ref: no)	-	-	-	-	-	-
yes	0.96	(0.94,0.98)	0.95	(0.92,0.97)	0.96	(0.93,0.98)
Other neurological disorders (ref: no)	-	-	-	-	-	-
yes	1.18	(1.14,1.22)	1.22	(1.18,1.26)	1.18	(1.15,1.22)
Chronic pulmonary disease (ref: no)	-	-	-	-	-	-
yes	0.96	(0.94,0.98)	0.96	(0.94,0.98)	0.97	(0.95,0.99)
Diabetes (uc) (ref: no)	-	-	-	-	-	-
yes	0.99	(0.97,1.02)	0.97	(0.94,1.00)	1.00	(0.97,1.02)
Diabetes (c) (ref: no)	-	-	-	-	-	-

yes	1.05	(1.02,1.08)	1.05	(1.02,1.08)	1.04	(1.01,1.07)
Renal failure (ref: no)	-	-	-	-	-	-
yes	1.13	(1.10,1.15)	1.19	(1.16,1.22)	1.15	(1.12,1.18)
Liver disease (ref: no)	-	-	-	-	-	-
yes	0.99	(0.97,1.01)	1.01	(0.99,1.04)	1.00	(0.98,1.02)
Blood loss anemia (ref: no)	-	-	-	-	-	-
yes	1.06	(1.00,1.12)	1.09	(1.02,1.16)	1.08	(1.02,1.15)
Deficiency anemia (ref: no)	-	-	-	-	-	-
yes	1.03	(1.00,1.06)	1.05	(1.02,1.09)	1.03	(0.99,1.06)
Alcohol abuse (ref: no)	-	-	-	-	-	-
yes	1.15	(1.11,1.20)	1.22	(1.17,1.27)	1.16	(1.12,1.21)
Drug abuse (ref: no)	-	-	-	-	-	-
yes	1.07	(1.01,1.14)	1.06	(0.99,1.14)	1.07	(1.00,1.14)
Other oncological disease (ref: no)	-	-	-	-	-	-
yes	0.83	(0.81,0.85)	0.85	(0.83,0.87)	0.85	(0.83,0.87)
Distant metastasis (ref: no)	-	-	-	-	-	-
yes	2.26	(2.21,2.30)	2.39	(2.33,2.45)	2.31	(2.26,2.36)
Hospital beds (ref: 1-299)	-	-	-	-	-	-
300-499	0.95	(0.92,0.99)	0.94	(0.91,0.98)	0.96	(0.92,1.00)
500-999	0.89	(0.85,0.93)	0.86	(0.82,0.90)	0.89	(0.85,0.93)
1000+	0.81	(0.76,0.87)	0.79	(0.73,0.84)	0.82	(0.77,0.88)
Teaching hospital (ref: no)	-	-	-	-	-	-
yes	0.96	(0.93,0.99)	0.94	(0.91,0.98)	0.95	(0.92,0.99)
University hospital (ref: no)	-	-	-	-	-	-
yes	0.81	(0.75,0.89)	0.79	(0.72,0.87)	0.82	(0.75,0.89)
Hospital ownership (ref: public)	-	-	-	-	-	-
non-profit	1.00	(0.96,1.03)	0.99	(0.95,1.02)	1.00	(0.97,1.04)
private	1.01	(0.97,1.06)	1.01	(0.97,1.06)	1.02	(0.98,1.06)
Number of patients	45,318		45,318		40,462	
Number of hospitals	1,051		1,051		1,043	
SD(RE)	0.16		0.16		0.15	
